# Outcome of necrotizing fasciitis and Fournier's gangrene with and without hyperbaric oxygen therapy: a retrospective analysis over 10 years

**DOI:** 10.1186/s13017-022-00448-6

**Published:** 2022-08-05

**Authors:** Assen Mladenov, Katharina Diehl, Oliver Müller, Christian von Heymann, Susanne Kopp, Wiebke K. Peitsch

**Affiliations:** 1 Department of Plastic, Reconstructive and Aesthetic Surgery - Center of Wound Medicine, Vivantes Klinikum im Friedrichshain, Berlin, Germany; 2 Faculty of Medicine, Charité - University Medicine, Berlin, Germany; 3 Mannheim Institute of Public Health, Social and Preventive Medicine, Heidelberg University, Mannheim, Germany; 4 Department of Medical Informatics, Biometry and Epidemiology, Professorship of Epidemiology and Public Health, Friedrich-Alexander-Universität Erlangen-Nürnberg (FAU), Erlangen, Germany; 5 Center of Hyperbaric Oxygen Therapy and Diving Medicine, Vivantes Klinikum im Friedrichshain, Berlin, Germany; 6 Department of Anaesthesiology, Intensive Care Medicine, Emergency Care Medicine and Pain Therapy, Vivantes Klinikum im Friedrichshain, Berlin, Germany; 7 Department of Dermatology and Phlebology, Vivantes Klinikum im Friedrichshain, Berlin, Germany

**Keywords:** Fournier's gangrene, Debridement, Hyperbaric oxygen therapy, Necrotizing fasciitis, Necrotizing soft tissue infections, Vacuum-assisted wound closure

## Abstract

**Background:**

Necrotizing soft tissue infections (NSTI) require immediate radical debridement, broad-spectrum antibiotics and intensive care. Hyperbaric oxygen therapy (HBOT) may be performed adjunctively, but unequivocal evidence for its benefits is still lacking.

**Methods:**

We performed a retrospective single-center study including 192 patients with necrotizing fasciitis or Fournier's gangrene to assess in-hospital mortality and outcome dependent on patient, disease and treatment characteristics with or without HBOT.

**Results:**

The in-hospital mortality rate was 27.6%. Factors associated with increased mortality according to multivariate analysis were higher age, affection of multiple or problem localizations (odds ratio (OR) = 2.88, *P* = 0.003), ineligibility for HBOT despite clinical indication (OR = 8.59, *P* = 0.005), pathogens in blood cultures (OR = 3.36, *P* = 0.002), complications (OR = 10.35, *P* < 0.001) and sepsis/organ dysfunction (OR = 19.58, *P* < 0.001). Factors associated with better survival included vacuum-assisted wound closure (OR = 0.17, *P* < 0.001), larger number of debridements (OR = 0.83, *P* < 0.001) and defect closure with mesh graft (OR = 0.06, *P* < 0.001) or flap (OR = 0.09, *P* = 0.024). When participants were stratified into subgroups without requirement of HBOT (*n* = 98), treated with HBOT (*n* = 83) and ineligible for HBOT due to contraindications (*n* = 11), the first two groups had similar survival rates (75.5% vs. 73.5%) and comparable outcome, although patients with HBOT suffered from more severe NSTI, reflected by more frequent affection of multiple localizations (*P* < 0.001), sepsis at admission (*P* < 0.001) and intensive care treatment (*P* < 0.001), more debridements (*P* < 0.001) and a larger number of antibiotics (*P* = 0.001). In the subgroup ineligible for HBOT, survival was significantly worse (36.4%, *P* = 0.022).

**Conclusion:**

These results point to a benefit from HBOT for treatment of NSTI in critically ill patients.

**Supplementary Information:**

The online version contains supplementary material available at 10.1186/s13017-022-00448-6.

## Background

Necrotizing soft tissue infections (NSTI) are rare, live-threatening diseases associated with a severe systemic inflammatory cascade triggered by bacterial toxins and with a high risk of mortality [1]. Two major types are necrotizing fasciitis (NF) and Fournier’s gangrene (FG), which occur in mono- and polymicrobial variants. Irrespective of the etiology and pathogen spectrum, NSTI require immediate and extensive surgical debridement, broad-spectrum antibiotics and intense supportive care. Hyperbaric oxygen therapy (HBOT) constitutes an adjunct treatment, the beneficial effects of which are attributable to high oxygen gradients in the tissues as a result of breathing 100% oxygen under 1.5–3 times the atmospheric pressure. Hyperoxygenation decelerates the infectious progress by improving the function of neutrophilic granulocytes and reduces tissue oedema [2]. Reactive oxygen species exert bacteriostatic and bactericidal effects on aerobes and anaerobes. Furthermore, high oxygen levels ameliorate tissue regeneration and improve the efficacy of antibiotics, especially in biofilm forming microbes [3]. However, evidence for the benefits of HBOT in terms of survival and clinical outcome of patients with NF and FG is still limited [4, 5].

The aim of our study was to assess in-hospital mortality and outcome of patients with NF and FG treated in our center in the last decade and to analyze the effects of patient, disease and treatment-related factors, particularly HBOT, on these parameters. For the latter, patients were stratified into three groups, one without requirement of HBOT, one treated with HBOT and one ineligible for HBOT despite clinical indication.

## Patients and methods

### Study cohort

All patients with NF and FG treated in the Department of Plastic, Reconstructive and Aesthetic Surgery - Center of Wound Medicine of the Vivantes Klinikum im Friedrichshain in Berlin, Germany between January 01, 2010, and October 01, 2020, were included into the study. Patients were identified by searching the electronic patient administration software (Orbis®, Agfa HealthCare, Bonn, Germany) for the diagnostic codes M72.6 (NF), N49.8 (FG in men) and N76.8 (FG in women) according to the International Classification of Diseases, Tenth Revision (ICD-10). Medical records of all patients were reviewed and checked for fulfillment of study criteria. Inclusion criteria were diagnosis of NF or FG according to clinical, laboratory, intraoperative and histological findings and final diagnosis of NF or FG documented in the records. Exclusion criteria were other NSTI such as gas gangrene, which is already a reimbursed indication for HBOT in Germany, and initially suspected NF or FG which was not confirmed.

The study was approved by the Ethics Committee of the Faculty of Medicine of Charité University Medicine Berlin (EA2/296/20). It was performed accordant with STROBE guidelines and compliant with the principles of the Declaration of Helsinki.

### Hyperbaric oxygen therapy

The indication and eligibility for HBOT were determined by an interdisciplinary team of plastic surgeons, anesthesiologists and intensive care specialists according to the Undersea and Hyperbaric Medical Society recommendations [6] and the European consensus conference criteria on hyperbaric medicine [7]. All patients in whom fascial or subfascial tissue destruction was seen intraoperatively and unambiguously vital margins could not be achieved during initial or subsequent debridements or who experienced circulatory deterioration despite radical debridement in the absence of other causes were considered for HBOT. The only absolute contraindication was untreated pneumothorax. Relative contraindications were chemotherapy with bleomycin, cisplatin or doxorubicin, severe hemodynamic instability and until October 2017 morbid obesity.

When deciding for HBOT, intraoperative tympanic paracentesis was carried out. The first HBOT session was administered immediately after debridement under controlled ventilation. Programmed redebridements were scheduled in 24-h intervals until a stabilization of the wound and cessation of tissue necrosis were seen.

Until October 2017, HBOT was performed in a HAUX Starmed 2200/5,5 Multiplace Chamber that was situated in a separate building in a distance of 200 m from the operating room (OR) and the intensive care unit (ICU). Patients had to be transferred on a stretcher in an emergency ambulance. The chamber could not accommodate an entire ICU bed because of a narrow entry door. This implied certain restrictions in patient selection. In particular, morbid obesity and severe hemodynamic instability prevented transport to and accommodation in the chamber in some cases. In October 2017, a new HAUX Starmed Quadro 300–2300/3,3/ICU Multiplace Chamber was put in operation, which is located in-house in 100 m distance from the OR and the ICU and accessible by elevator. Patient transfer into another bed or stretcher is not required any more, as the patients remain in an HBOT-compatible ICU bed. Therefore, morbid obesity ceased to pose a restriction for HBOT. Since the start-up of the new chamber the subgroup of patients ineligible for HBOT is confined to the most critically instable patients, critically ill patients with intention to best supportive care and patients with untreated pneumothorax.

The hyperbaric chambers are affiliated with the Department of Intensive Care Medicine and provide full intensive care equipment. They are staffed with a highly experienced interdisciplinary team around the clock, and patients are monitored by an intensive care specialist throughout the treatment sessions. The standard treatment regimen consists of three dives at a maximum pressure of 300 kPa with a duration of 90 min (TS 300/90) within the first 24 h, followed by a minimum of two further treatments according to TS 240/90 until a stop of disease progression. The treatment algorithm was described in detail by Schmale and colleagues [8].

### Data collection

The following patient, disease and treatment characteristics were documented as pseudonymized data: sex, age at admission, transfer from another hospital, diagnosis of NF or FG, disease localization (for categories see Table 1), clinical signs and symptoms at admission, pain (mild/none, intermediate, strong), laboratory parameters at admission (leucocyte count, hemoglobin, C-reactive protein, creatinine, sodium, serum glucose, fibrinogen), pathogen growth in blood cultures collected prior to antibiotic treatment, sepsis according to Sepsis-2 criteria [9] and/or the quick sequential organ failure assessment (qSOFA) score, Laboratory Risk Indicator for NECrotizing fasciitis (LRINEC), modified LRINEC [10], pathogens in wound swabs, tissue samples, urine and stool, histological evidence of fascial involvement, number and kind of comorbidities, number of surgical debridements, time to first debridement (h), vacuum-assisted closure (VAC; yes/no), number of VAC changes, HBOT (no, not indicated vs. yes vs. no, ineligible due to contraindications), number of HBOT cycles, defect coverage, kind and number of antibiotics, antibiotic groups and changes, duration of hospitalization (days (d)), ICU stay (if yes, duration (d)), and complications (for categories, see Tables 2, 4).

**Table 1:** Relation of patient and disease characteristics with mortality

Characteristic	Total, *n* (%)	Mortality, *n* (%)		
		Yes	No	*P*^a^
	192 (100)	53 (27.6)	139 (72.4)	
Sex				
Male	127 (66.1)	34 (64.2)	93 (66.9)	0.718
Female	65 (33.9)	19 (35.8)	46 (33.1)	
Age (years)				
Mean (SD)	61.2 (15.1)	**65.7 (14.1)**	**59.5 (15.2)**	**0.006**
< 60	87 (45.3)	**15 (28.3)**	**72 (51.8)**	**0.003**
≥ 60	105 (54.7)	**38 (71.7)**	**67 (48.2)**	
Diagnosis				
Necrotizing fasciitis	153 (79.7)	44 (83.0)	109 (78.4)	0.479
Fournier's gangrene	39 (20.3)	9 (17.0)	30 (21.6)	
Localization				
Lower extremity	137 (71.4)	39 (73.6)	98 (70.5)	0.673
Genital/perineal/gluteal	74 (38.5)	24 (45.3)	50 (36.0)	0.236
Trunk	40 (20.8)	14 (26.4)	26 (18.7)	0.240
Retroperitoneal^b^	4 (2.1)	3 (5.7)	1 (0.7)	0.065
Upper extremity^b^	10 (5.2)	3 (5.7)	7 (5.0)	1.000
Head/neck	1 (0.5)	1 (100.0)	0 (0.0)	n.d
Multiple localizations	56 (29.2)	**23 (43.4)**	**33 (23.7)**	**0.007**
Initial signs and symptoms				
Sepsis at admission	132 (68.8)	41 (77.4)	91 (65.5)	0.112
Positive blood culture^c^	69 (44.8)	**30 (65.2)**	**39 (36.1)**	**0.001**
Leucocyte count, mean (SD)	18.4 (8.3)	19.3 (9.8)	18.0 (7.7)	0.454
CRP, mean (SD)	259.4 (138.4)	236.7 (141.0)	268.3 (136.8)	0.156
LRINEC^d^, mean (SD)	7.1 (2.8)	7.3 (2.9)	7.1 (2.8)	0.556
Modified LRINEC^e^, mean (SD)	10.2 (3.3)	10.4 (3.4)	10.2 (3.3)	0.754
Comorbidities				
Number, mean (SD)	2.8 (1.6)	3.0 (1.5)	2.7 (1.6)	0.206
< 3	95 (49.5)	**20 (37.7)**	**75 (54.0)**	**0.044**
≥ 3	97 (50.5)	**33 (62.3)**	**64 (46.0)**	
Diabetes mellitus	93 (48.4)	27 (50.9)	66 (47.5)	0.668
Vascular diseases^f^	58 (30.2)	16 (30.2)	42 (30.2)	0.997
Renal diseases	52 (27.1)	16 (30.2)	36 (25.9)	0.550
Cardiac diseases	75 (39.1)	25 (47.2)	50 (36.0)	0.155
Liver diseases	32 (16.7)	**15 (28.3)**	**17 (12.2)**	**0.008**
Arterial hypertension	115 (59.9)	26 (49.1)	89 (64.0)	0.058
History of stroke	15 (7.8)	5 (9.4)	10 (7.2)	0.605
Alcohol abuse	29 (15.1)	8 (15.1)	21 (15.1)	0.998
Substance abuse (i.v.)^b^	10 (5.2)	5 (9.4)	5 (3.6)	0.142
Psychiatric diseases^b^	17 (8.9)	2 (3.8)	15 (10.8)	0.161
Malignant diseases	29 (15.1)	12 (22.6)	17 (12.2)	0.072
Immunosuppression	28 (14.6)	12 (22.6)	16 (11.5)	0.051

For calculation of percentages, the number of patients in each column (i.e., in the total cohort and in subgroups with and without mortality) was set to 100%

*CRP* C-reactive protein, *i.v.* intravenous, *n.d.* not determined, *SD* standard deviation

^a^Characteristics of subgroups with and without mortality were compared with Chi-squared test for categorical variables, with Mann-Whitney-U test for continuous variables in two categories and with Kruskal-Wallis test for linear variables in three categories. Significant differences are highlighted in bold

^b^Exact Fisher-t-test

^c^Blood cultures were taken before initiation of antibiotics

^d^LRINEC: Laboratory Risk Indicator for NECrotizing fasciitis

^e^The modified LRINEC could be calculated in 131 participants (*n* = 61: missing data)

^f^Vascular diseases comprised peripheral arterial occlusive disease, chronic venous insufficiency, deep vein thrombosis and pulmonary embolism

**Table 2:** Relation of treatment characteristics with mortality

Characteristic	Total, *n* (%)	Mortality, *n* (%)		
		Yes	No	*P*^a^
	192 (100)	53 (27.6)	139 (72.4)	
Surgical treatment				
Debridements,^b^ mean (SD)	7.5 (5.3)	**5.2 (4.2)**	**8.3 (5.5)**	** < 0.001**
≤ 5	82 (42.7)	**32 (60.4)**	**50 (36.0)**	**0.002**
> 5	110 (57.3)	**21 (39.6)**	**89 (64.0)**	
Time to 1st debrid. ≤ 12 h^c^	92 (47.9)	23 (43.4)	69 (49.6)	0.439
Time to 1st debrid. ≤ 24 h	115 (59.9)	27 (50.9)	88 (63.3)	0.118
VAC therapy	120 (62.5)	**17 (32.1)**	**103 (74.1)**	** < 0.001**
VAC changes, mean (SD)	5.3 (4.0)	4.9 (2.6)	5.4 (4.2)	0.855
≤ 5	82 (68.3)	9 (52.9)	73 (70.9)	0.141
> 5	38 (31.7)	8 (47.1)	30 (29.1)	
Defect closure	135 (70.3)	**13 (24.5)**	**122 (87.8)**	** < 0.001**
Mesh graft	109 (56.8)	**8 (15.1)**	**101 (72.7)**	** < 0.001**
Flap	27 (14.1)	**1 (1.9)**	**26 (18.7)**	**0.003**
Amputation^d^	46 (24.0)	17 (32.1)	29 (20.9)	0.104
Secondary healing	37 (19.3)	6 (11.3)	31 (22.3)	0.085
HBOT				
No, not indicated	98 (51.1)	**24 (45.3)**	**74 (53.2)**	**0.022**
Yes	83 (43.2)	**22 (41.5)**	**61 (43.9)**	
No, ineligible^e^	11 (5.7)	**7 (13.2)**	**4 (2.9)**	
Antibiotic therapy				
Number of AB, mean (SD)	4.6 (2.2)	4.3 (2.2)	4.8 (2.2)	0.153
≤ 3	67 (34.9)	24 (45.3)	43 (30.9)	0.062
> 3	125 (65.1)	29 (54.7)	96 (69.1)	
AB groups, mean (SD)	3.3 (1.3)	3.3 (1.5)	3.3 (1.3)	0.885
≤ 3	110 (57.3)	29 (54.7)	81 (58.3)	0.656
> 3	82 (42.7)	24 (45.3)	58 (41.7)	
Changes of AB, mean (SD)	2.5 (2.1)	**2.0 (2.0)**	**2.7 (2.0)**	**0.007**
≤ 2	115 (59.9)	37 (69.8)	78 (56.1)	0.083
> 2	77 (40.1)	16 (30.2)	61 (43.9)	
Hospital treatment				
ICU treatment	172 (89.6)	**52 (98.1)**	**120 (86.3)**	**0.017**
Days in ICU, mean (SD)	13.3 (16.1)	13.4 (16.8)	13.2 (16.0)	0.815
0 days	20 (10.4)	**1 (1.9)**	**19 (13.7)**	**0.012**
1–7 days	82 (42.7)	**30 (56.6)**	**52 (37.4)**	
> 7 days	90 (46.9)	**22 (41.5)**	**68 (48.9)**	
Days in hospital, mean (SD)^f^	40.2 (26.3)	**22.8 (25.1)**	**46.8 (23.7)**	** < 0.001**
≤ 40 days	109 (56.8)	**40 (75.5)**	**69 (49.6)**	**0.001**
> 40 days	83 (43.2)	**13 (24.5)**	**70 (50.4)**	
Complications				
Total	120 (63.5)	**47 (92.2)**	**73 (52.9)**	** < 0.001**
Infections^g^	95 (49.5)	25 (47.2)	70 (50.4)	0.693
Sepsis/organ dysfunction^h^	76 (39.6)	**45 (84.9)**	**31 (22.3)**	** < 0.001**
Impaired wound healing	46 (24.0)	**5 (9.4)**	**41 (29.5)**	**0.004**
Prolonged delirium	30 (15.6)	4 (7.5)	26 (18.7)	0.057
Stump complications	14 (7.3)	2 (3.8)	12 (8.6)	0.247
Decubitus	9 (4.7)	1 (1.9)	8 (5.8)	0.257
Thrombosis	9 (4.7)	2 (3.8)	7(5.0)	0.711
Anus praeter^i, j^	29 (15.1)	5 (9.4)	24 (17.3)	0.259
Ileus^i^	4 (2.1)	2 (3.8)	2 (1.4)	0.306
Stroke^i^	3 (1.6)	1 (1.9)	2 (1.4)	1.000
Cardiac arrest^i^	3 (1.6)	2 (3.8)	1 (0.7)	0.185
Other complications^i, k^	11 (5.7)	5 (9.4)	6 (4.3)	0.180

For calculation of percentages, the number of patients in each column (i.e., in the total cohort and in subgroups with and without mortality) was set to 100%

*AB* antibiotics, *debrid*. Debridement, *HBOT* hyperbaric oxygen therapy, *ICU* intensive care unit, *SD* standard deviation, *VAC* vacuum-assisted closure

^a^Characteristics of subgroups with and without mortality were compared with Chi-squared test for categorical variables and with Mann–Whitney-U test for continuous variables. Significant differences are highlighted in bold

^b^Debridements included both radical debridements and minor surgical debridements for wound conditioning performed in our hospital and/or in other hospitals or departments prior to admission to our center

^c^Time to first debridement was calculated from the time of admission to our clinic until debridement

^d^In the subgroup with amputation, *n* = 2 patients (1.0%) obtained a minor amputation, *n* = 3 (1.6%) a transtibial amputation, *n* = 3 (1.6%) a knee disarticulation, *n* = 34 (17.7%) a transfemoral amputation, *n* = 5 (1.6%) a hip disarticulation and *n* = 8 (4.2%) an atypical amputation. 18 patients (9.4%) had a reamputation

^e^*n* = 11 patients were ineligible for HBOT due to factors specified in Additional file 1: Table S1

^f^Defined as number of days from admission to our or to an external hospital until discharge from our hospital or death

^g^Infections comprised externally acquired and nosocomial urinary tract infections (*n* = 63), Clostridium difficile infections (*n* = 28), pneumonia (*n* = 9), wound infections (*n* = 3) and catheter sepsis (*n* = 23)

^h^The category "sepsis/organ dysfunction" contained patients with sepsis and/or subsequent renal failure and/or failure of additional organs

^i^Exact Fisher-t-test

^j^In 29 patients (*n* = 14 with necrotizing fasciitis and *n* = 15 with Fournier’s gangrene), colostomy was performed prior to admission to our department

^k^Other complications comprised acute coronary syndrome (*n* = 1), gastrointestinal bleeding (*n* = 3), requirement of resuscitation (*n* = 2), tumor progression (*n* = 1), hypoxic brain damage (*n* = 1), hematuria (*n* = 1), stoma complications (*n* = 1) and biliary stent occlusion (*n* = 1)

Co-primary outcome criteria were in-hospital mortality, i.e., mortality in the time between admission to our or another hospital and death or discharge from our hospital, in the total cohort and in HBOT subgroups. Secondary outcome parameters were impairment in survivors (none vs. mild-moderate vs. severe) and return to the previous living environment without severe disabling impairment (yes/no, for definition, see Table 4).

### Statistical analysis

Statistical analyses were performed with IBM SPSS Statistics 25. For subgroup analyses, patients were stratified according to sex, age, NF or FG, disease localization, number of comorbidities, debridements and VAC changes, HBOT (no, not indicated vs. yes vs. no, ineligible), number of antibiotics, antibiotic groups and changes of antibiotics, duration of ICU treatment and of hospitalization (for categories, see Tables 1–4). Associations between binary and categorical patient, disease and treatment characteristics and mortality were tested for significance with Pearson's Chi-squared test or with Fisher's exact test when the conditions for the Chi-squared test were not fulfilled (expected frequencies < 5). Nonparametric continuous variables were analyzed with Mann-Whitney U test.

To examine associations between patient, disease and treatment characteristics and HBOT, the cohort was subdivided into three groups (HBOT no, not indicated vs. yes vs. no, ineligible, due to factors specified in Additional file 1: Table S1). Chi-squared test and Fisher’s exact test were used for binary and categorical variables and Kruskal-Wallis test for continuous variables.

Cumulative survival of the HBOT subgroups from admission to our or another hospital to death or discharge from our hospital was calculated with the Kaplan–Meier method and graphically displayed by Kaplan–Meier curves. Differences were tested for significance with the log-rank test with two degrees of freedom.

Furthermore, we calculated multivariate logistic regression models with mortality as the dependent variable. The baseline model contained sex, age, problem localization (defined as localization in the retroperitoneal area, head/neck region and/or in multiple localizations; yes/no), number of comorbidities and the LRINEC as independent variables. In models 1–13, one additional parameter at a time was integrated into the baseline model. Results were expressed as odds ratio (OR) with 95% confidence intervals (CIs).

A p-value < 0.05 was considered statistically significant in all analyses.

## Results

### Patient and disease characteristics and their impact on mortality

Out of 240 patients with diagnostic codes of NSTI, 192 fulfilled study criteria. All of these were included. Two thirds were male, and the mean age was 61.2 years (Table 1). 78.6% were referred from other departments or hospitals, and 32.3% had received a debridement prior to transfer. Further baseline characteristics are summarized in Table 1.

The overall in-hospital mortality rate was 27.6% without significant differences between patients directly admitted or transferred to our center. Patients who died were significantly older than survivors (*P* = 0.006). The most common localization was the lower extremity (71.4%), followed by the perianal/genital/gluteal area (38.5%). Affection of multiple localizations was documented in 29.2% and associated with a higher risk of mortality (*P* = 0.007).

**Fig. 1:**
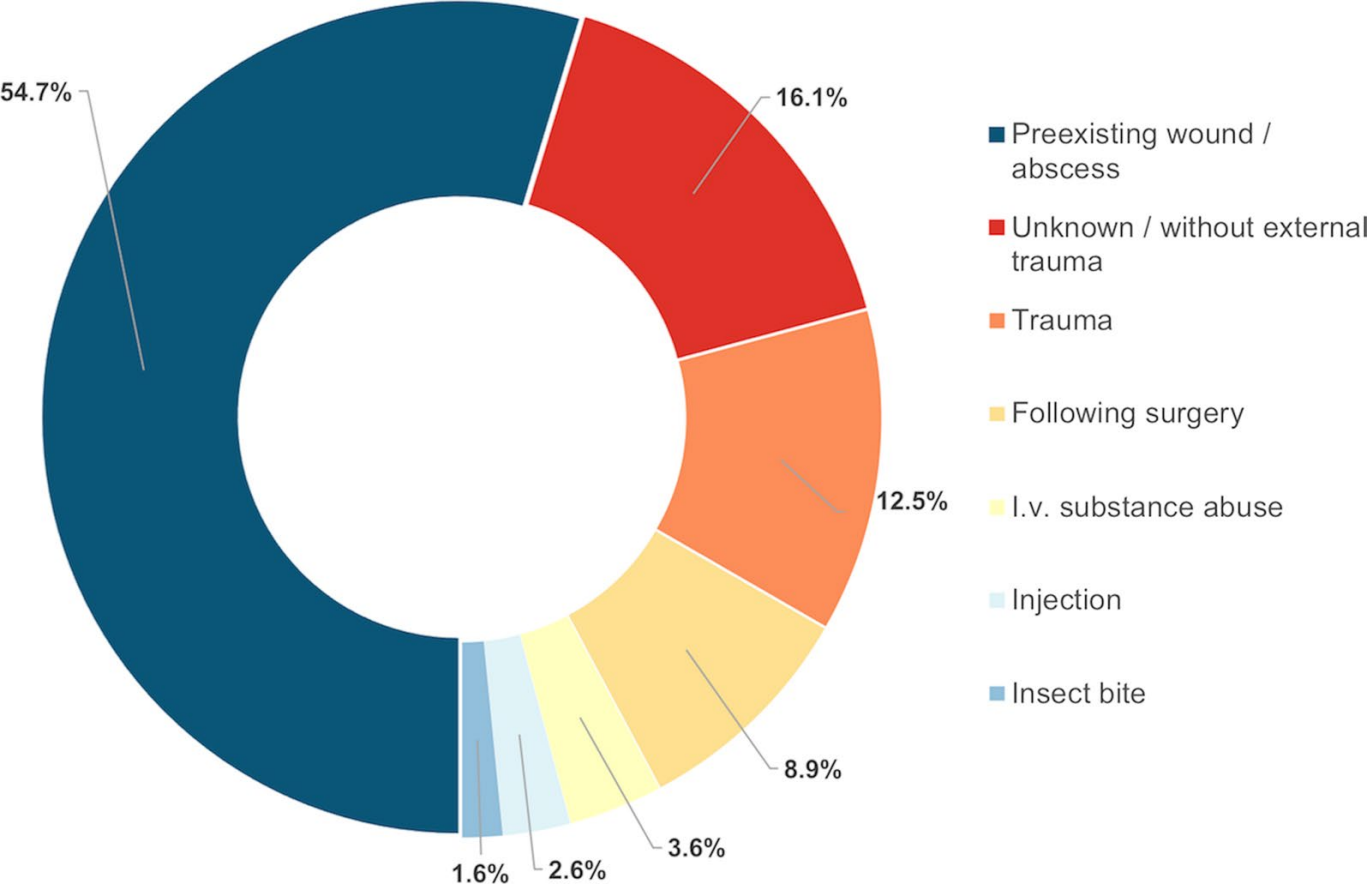
Etiology of necrotizing fasciitis and Fournier's gangrene

**Fig. 2:**
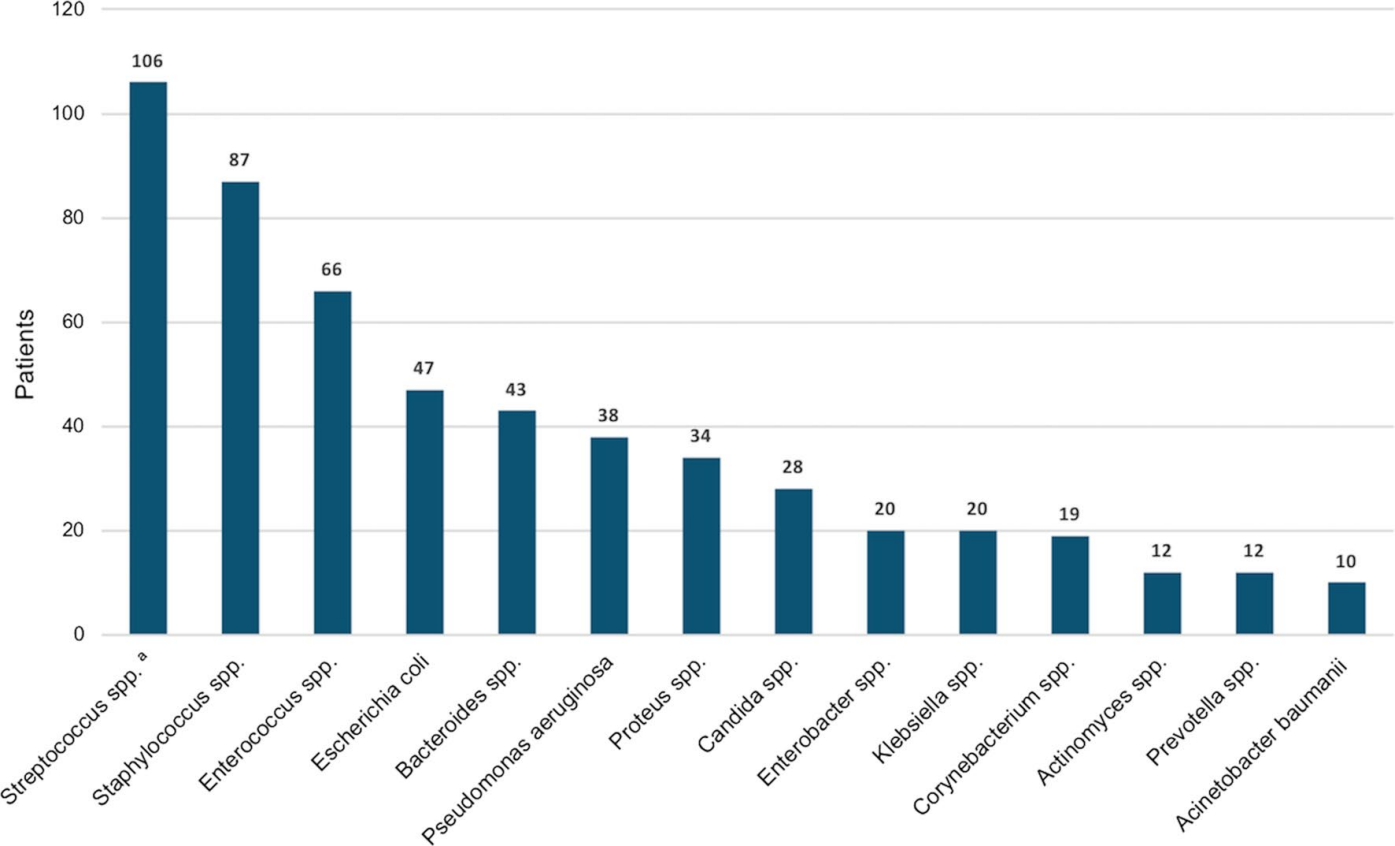
Spectrum of pathogens detected in wound swabs and/or tissue specimens. ^a^Streptococcus spp. comprised most frequently S. pyogenes (*n* = 29), S. dysgalactiae (*n* = 18), S. anginosus (*n* = 12) and S. agalactiae (*n* = 9). Other pathogens were detected in 81 patients, most frequently Stenotrophomonas maltophilia (*n* = 7), Serratia spp. (*n* = 6), Coagulase-negative staphylococci (*n* = 5), Citrobacter spp. (*n* = 5) and Fusobacterium spp. (*n* = 4)

NSTI occurred most frequently on preexisting wounds (54.7%; Fig. 1). The spectrum of pathogens isolated from wound swabs and/or tissue specimens is shown in Fig. 2. Polymicrobial pathogenesis (NSTI type I) was most common (68.8%). Pathogens in blood cultures were detected in 44.8% and associated with higher mortality (*P* = 0.001; Table 1).

Almost all participants (92.2%) suffered from comorbidities, most frequently from hypertension (59.9%) and diabetes (48.4%, Table 1). Patients with ≥ 3 comorbidities (*P* = 0.044) or liver disease (*P* = 0.008) were more likely to have a fatal outcome than others.

### Treatments

All patients obtained at least one surgical debridement, 47.9% within 12 h and 59.9% within 24 h after admission (Table 2). Survivors had a larger number of debridements when considering both radical and minor procedures for wound conditioning (*P* < 0.001). VAC therapy was administered in 62.5% and associated with a higher chance of survival (*P* < 0.001), similar as defect closure with mesh graft (*P* < 0.001) or flaps (*P* = 0.003). Failure to achieve wound closure correlated with a higher risk of mortality (*P* < 0.001). Amputation was necessary in 24.0%, orchiectomy in 3.6%.

43.2% of the patients underwent HBOT, whereas in 51.1% HBOT was not indicated. Mortality rates were similar in both groups (26.5% vs. 24.5%; Table 4). Eleven patients (5.7%) were ineligible for HBOT due to contraindications specified in Additional file 1: Table S1. These patients were significantly more likely to die than both other groups (mortality rate 63.6%; *P* = 0.022; Tables 2, 4).

**Fig. 3:**
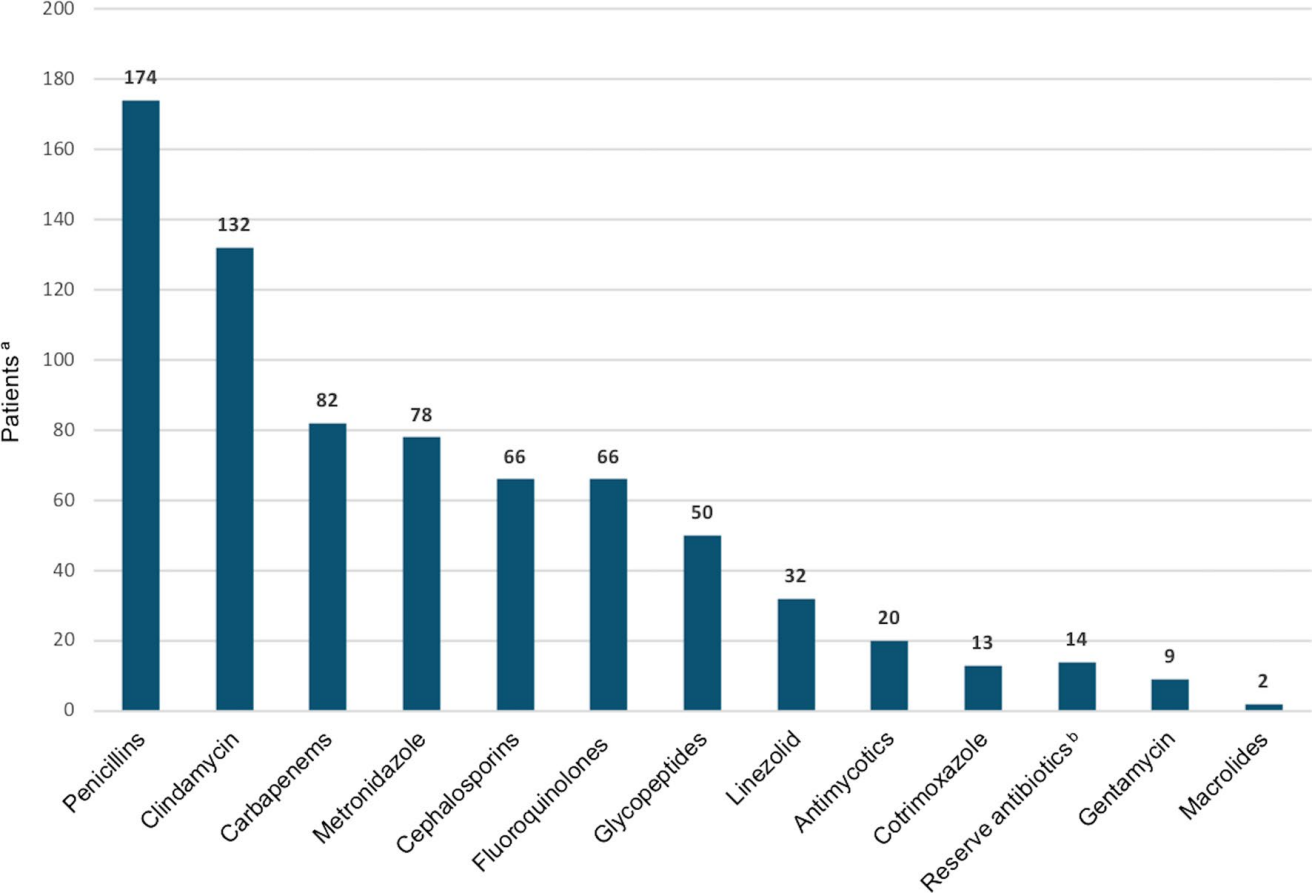
Frequently administered antibiotics. ^a^Number of patients receiving each class of antibiotic. ^b^Reserve antibiotics, i.e., antibiotics of last resort used with strict indication, included colistin, daptomycin and tigecycline

Frequently administered antibiotics are shown in Fig. 3. The mean number of antibiotics applied in each participant was 4.6. Survivors underwent more antibiotic changes than patients who died (*P* = 0.007; Table 2).

The vast majority of patients were treated in the ICU, among them virtually all who died (98.1% vs. 86.3% of the survivors, *P* = 0.017). The total duration of the hospital stay was significantly longer in survivors (Table 2).

Complications of any kind occurred in 63.5%, more frequently in patients with fatal outcome (*P* < 0.001; Table 2). In particular, sepsis and organ dysfunction were more common in patients who died (*P* < 0.001), whereas impaired wound healing occurred more frequently in survivors (*P* = 0.004).

### Subgroup analyses according to HBOT

Patients ineligible for HBOT were younger than both other HBOT subgroups (*P* = 0.008 in three-group comparison; Table 3). The subgroups treated with HBOT and ineligible for HBOT suffered more frequently from NSTI in the genital/inguinal/perineal area (*P* = 0.033), on the trunk (*P* < 0.001) and in multiple localizations (*P* < 0.001) than patients without indication for HBOT. Furthermore, these two subgroups had significantly higher mean CRP values, leucocyte counts, LRINEC and modified LRINEC scores and rates of sepsis at admission than the subgroup without requirement of HBOT (Table 3).

**Table 3:** Patient and disease characteristics of subgroups with and without hyperbaric oxygen therapy (HBOT)

Characteristic	HBOT			
	No, not indicated	Yes	No, ineligible	*P*^a^
	*n* (%)	*n* (%)	*n* (%)	
Total (*n* = 192)	98 (51.0)	83 (43.2)	11 (5.7)	
Sex				
Male	**69 (70.4)**	**48 (57.8)**	**10 (90.9)**	**0.041**
Female	**29 (29.6)**	**35 (42.2)**	**1 (9.1)**	
Age (years)				
Mean (SD)	**64.3 (14.1)**	**58.8 (15.3)**	**52.3 (16.8)**	**0.008**
< 60	**35 (35.7)**	**44 (53.0)**	**8 (72.7)**	**0.011**
≥ 60	**63 (64.3)**	**39 (47.0)**	**3 (27.3)**	
Diagnosis				
Necrotizing fasciitis	78 (79.6)	64 (77.1)	11 (100.0)	0.207
Fournier's gangrene	20 (20.4)	19 (22.9)	0 (0.0)	
Localization				
Lower extremity	76 (77.6)	53 (63.9)	8 (72.7)	0.127
Genital/inguinal/perineal	**29 (29.6)**	**40 (48.2)**	**5 (45.5)**	**0.033**
Trunk	**9 (9.2)**	**26 (31.3)**	**5 (45.5)**	** < 0.001**
Retroperitoneal^b^	1 (1.0)	3 (3.6)	0 (0.0)	0.421
Upper extremity^b^	2 (2.0)	8 (9.6)	0 (0.0)	0.052
Head/neck	0 (0.0)	1 (1.2)	0 (0.0)	n.d
Multiple localizations	**16 (16.3)**	**35 (42.2)**	**5 (45.5)**	** < 0.001**
Initial signs and symptoms				
Sepsis at admission	**49 (50.0)**	**74 (89.2)**	**9 (81.8)**	** < 0.001**
Positive blood culture^c^	32 (46.4)	30 (40.0)	7 (70.0)	0.189
Leucocyte count, mean (SD)	**16.7 (7.7)**	**19.8 (8.2)**	**21.8 (11.4)**	**0.017**
CRP, mean (SD)	**235.6 (142.0)**	**284.2 (132.0)**	**284.5 (126.2)**	**0.049**
LRINEC^d^, mean (SD)	**6.6 (2.9)**	**7.6 (2.5)**	**8.3 (2.8)**	**0.015**
Modified LRINEC^e^, mean (SD)	**9.4 (3.4)**	**11.1 (2.8)**	**11.0 (4.8)**	**0.011**
Comorbidities				
Number, mean (SD)	3.0 (1.5)	2.5 (1.7)	2.6 (1.4)	0.066
< 3	**39 (39.8)**	**49 (59.0)**	**7 (63.6)**	**0.022**
≥ 3	**59 (60.2)**	**34 (41.0)**	**4 (36.4)**	
Diabetes mellitus	51 (52.0)	35 (42.2)	7 (63.6)	0.243
Vascular diseases^f^	36 (36.7)	19 (22.9)	3 (27.3)	0.127
Renal diseases	28 (28.6)	20 (24.1)	4 (36.4)	0.617
Cardiac diseases	44 (44.9)	28 (33.7)	3 (27.3)	0.219
Liver diseases	18 (18.4)	11 (13.3)	3 (27.3)	0.408
Arterial hypertension	**67 (68.4)**	**45 (54.2)**	**3 (27.3)**	**0.012**
History of stroke	7 (7.1)	8 (9.6)	0 (0.0)	0.502
Alcohol abuse	18 (18.4)	10 (12.0)	1 (9.1)	0.421
Substance abuse (i.v.)	3 (3.1)	6 (7.2)	1 (9.1)	0.380
Psychiatric diseases	6 (6.1)	11 (13.3)	0 (0.0)	0.138
Malignant diseases	16 (16.3)	10 (12.0)	3 (27.3)	0.370
Immunosuppression	17 (17.3)	10 (12.0)	1 (9.1)	0.523

For calculation of percentages, the number of patients in each column (i.e., HBOT no, not indicated, HBOT yes and HBOT no, ineligible) was set to 100%

*CRP* C-reactive protein, *i.v.* intravenous, *SD* standard deviation

^a^Characteristics of the three subgroups were compared with Chi-squared test for categorical variables and with Kruskal-Wallis test for continuous variables. Significant differences are highlighted in bold

^b^Exact Fisher-t-test

^c^Blood cultures were taken before initiation of antibiotics

^d^LRINEC: Laboratory Risk Indicator for NECrotizing fasciitis

^e^The modified LRINEC could be calculated in 131 participants (*n* = 61: missing data)

^f^Vascular diseases comprised peripheral arterial occlusive disease, chronic venous insufficiency, deep vein thrombosis and pulmonary embolism

HBOT patients received more debridements than both other subgroups (*P* < 0.001; Table 4). This subgroup and patients ineligible for HBOT were more likely to obtain their first debridement within 12 and 24 h after admission than patients without indication of HBOT (*P* < 0.001). Defect coverage was attempted less frequently in patients ineligible for HBOT (*P* = 0.040).

**Table 4:** Treatment characteristics, complications and outcome of patients with and without hyperbaric oxygen therapy (HBOT)

Characteristic	HBOT			
	No, not indicated	Yes	No, ineligible	*P*^a^
	*n* (%)	*n* (%)	*n* (%)	
Total (*n* = 192)	98 (51.0)	83 (43.2)	11 (5.7)	
Surgical treatment				
Debridements,^b^ mean (SD)	**6.0 (4.1)**	**9.1 (5.8)**	**7.9 (7.3)**	** < 0.001**
Time to 1st debrid. ≤ 12 h^c^	**33 (33.7)**	**51 (61.4)**	**8 (72.7)**	** < 0.001**
Time to 1st debrid. ≤ 24 h	**45 (45.9)**	**62 (74.7)**	**8 (72.7)**	** < 0.001**
VAC therapy	62 (63.3)	53 (63.9)	5 (45.5)	0.484
VAC changes, mean (SD)	4.6 (3.0)	5.9 (4.7)	8.0 (5.5)	0.062
Defect coverage	**71 (72.4)**	**60 (72.3)**	**4 (36.4)**	**0.040**
Mesh graft	58 (59.2)	48 (57.8)	3 (27.3)	0.124
Flap	12 (12.2)	14 (16.9)	1 (9.1)	0.597
Amputation	21 (21.4)	20 (24.1)	5 (45.5)	0.209
Secondary healing	14 (14.3)	20 (24.1)	3 (27.3)	0.196
Antibiotic therapy				
Number of AB, mean (SD)	**4.0 (1.9)**	**5.2 (2.2)**	**5.6 (2.9)**	**0.001**
≤ 3	**46 (46.9)**	**18 (21.7)**	**3 (27.3)**	**0.002**
> 3	**52 (53.1)**	**65 (78.3)**	**8 (72.7)**	
AB groups, mean (SD)	**3.0 (1.3)**	**3.6 (1.2)**	**3.6 (1.6)**	**0.006**
≤ 3	**67 (68.4)**	**39 (47.0)**	**4 (36.4)**	**0.005**
> 3	**31 (31.6)**	**44 (53.0)**	**7 (63.6)**	
Changes of AB, mean (SD)	2.4 (1.9)	2.6 (2.0)	3.2 (3.3)	0.795
≤ 2	63 (64.3)	45 (54.2)	7 (63.6)	0.374
> 2	35 (35.7)	38 (45.8)	4 (36.4)	
Hospital treatment				
ICU treatment	**79 (80.6)**	**82 (98.8)**	**11 (100.0)**	** < 0.001**
Days in ICU, mean (SD)	**8.2 (11.7)**	**18.2 (16.8)**	**21.4 (28.1)**	** < 0.001**
0 days	**19 (19.4)**	**1 (1.2)**	**0 (0.0)**	** < 0.001**
1–7 days	**52 (53.1)**	**24 (28.9)**	**6 (54.5)**	
> 7 days	**27 (27.6)**	**58 (69.9)**	**5 (45.5)**	
Days in hospital, mean (SD)^d^	39.0 (25.2)	42.1 (24.7)	35.6 (44.4)	0.203
≤ 40 days	59 (60.2)	43 (51.8)	7 (63.6)	0.469
> 40 days	39 (39.8)	40 (48.2)	4 (36.4)	
Infections	45 (45.9)	44 (53.0)	6 (54.5)	0.599
Urinary tract infection	25 (25.5)	35 (42.2)	3 (27.3)	0.054
C. difficile infection	17 (17.3)	10 (12.0)	1 (9.1)	0.523
Pneumonia	3 (3.1)	4 (4.8)	2 (18.2)	0.079
Wound infection	1 (1.0)	2 (2.4)	0 (0.0)	0.688
Catheter sepsis	7 (7.1)	13 (15.7)	3 (27.3)	0.058
Multiresistent pathogens^e^	**7 (100.0)**	**0 (0.0)**	**0 (0.0)**	**0.031**
Complications				
Total (*n* = 120)	54 (56.8)	57 (68.7)	9 (81.8)	0.113
Sepsis/organ dysfunction^f^	**32 (32.7)**	**35 (42.2)**	**9 (81.8)**	**0.006**
Impaired wound healing	22 (22.4)	21 (25.3)	3 (27.3)	0.873
Prolonged delirium	**10 (10.2)**	**20 (24.1)**	**0 (0.0)**	**0.013**
Stump complications	5 (5.1)	7 (8.4)	2 (18.2)	0.248
Decubitus	2 (2.0)	5 (6.0)	2 (18.2)	0.042
Thrombosis/embolism	4 (4.1)	3 (3.6)	2 (18.2)	0.092
Anus praeter	**6 (6.1)**	**20 (24.1)**	**3 (27.3)**	**0.002**
Ileus	3 (3.1)	1 (1.2)	0 (0.0)	0.604
Stroke	0 (0.0)	3 (3.6)	0.(0.0)	0.135
Cardiac arrest	3 (3.1)	0 (0.0)	0 (0.0)	0.232
Other complications^g^	6 (6.1)	5 (6.0)	0 (0.0)	0.701
Outcome				
Survived	**74 (75.5)**	**61 (73.5)**	**4 (36.4)**	**0.022**
No impairment	16 (21.6)	8 (13.1)	1 (25.0)	0.155
Mild-moderate impairment^h^	38 (51.4)	31 (50.8)	0 (0.0)	
Severe impairment^i^	20 (27.0)	22 (36.1)	3 (75.0)	
Return to former home^j^	**57 (58.2)**	**45 (54.2)**	**1 (9.1)**	**0.008**

For calculation of percentages, the number of patients in each column (i.e., HBOT no, not indicated, HBOT yes and HBOT no, ineligible) was set to 100%

*AB* antibiotics, *C difficile* Clostridium difficile, *debrid*. Debridement, *h* hours, *ICU* intensive care unit, *SD* standard deviation, *VAC* vacuum-assisted closure

^a^Characteristics of the three subgroups were compared with Chi-squared test for categorical variables and with Kruskal-Wallis test for continuous variables. Significant differences are highlighted in bold

^b^Debridements included both radical debridements and minor surgical debridements for wound conditioning

^c^Time to first debridement was calculated from the time of admission to our hospital until debridement

^d^Defined as number of days from admission to our or to an external hospital until discharge from our hospital or death

^e^Wound infection with multiresistant pathogens

^f^The category "sepsis/organ dysfunction" contained patients with sepsis and/or subsequent renal failure and/or failure of additional organs

^g^Other complications affecting *n* = 11 patients comprised acute coronary syndrome (*n* = 1), gastrointestinal bleeding (*n* = 3), requirement of resuscitation (*n* = 2), tumor progression (*n* = 1), hypoxic brain damage (*n* = 1), hematuria (*n* = 1), stoma complications (*n* = 1) and biliary stent occlusion (*n* = 1)

^h^Mild-moderate impairment included, e.g., remaining wound defects at discharge from hospital and temporary impairment of mobility

^i^Severe impairment comprised permanent loss of walking ability, permanent confinement to a wheelchair or to bed, incontinence, critical illness neuropathy, or post intensive care syndrome

^j^Return to the previous living environment without severe disabling impairment

The mean number of antibiotics was highest in the subgroup ineligible for HBOT, followed by the subgroup with HBOT (*P* = 0.001; Table 4). These two subgroups were also treated in the ICU more frequently (*P* < 0.001) and for a longer mean duration (*P* < 0.001) than patients without indication of HBOT. Sepsis and organ dysfunction were documented by far most frequently in patients ineligible for HBOT (81.8%, *P* = 0.006).

HBOT patients and patients without requirement of HBOT were more likely to survive (*P* = 0.022) and return to their previous living environment without severe disabling impairment (*P* = 0.008) than those ineligible for HBOT.

**Fig. 4:**
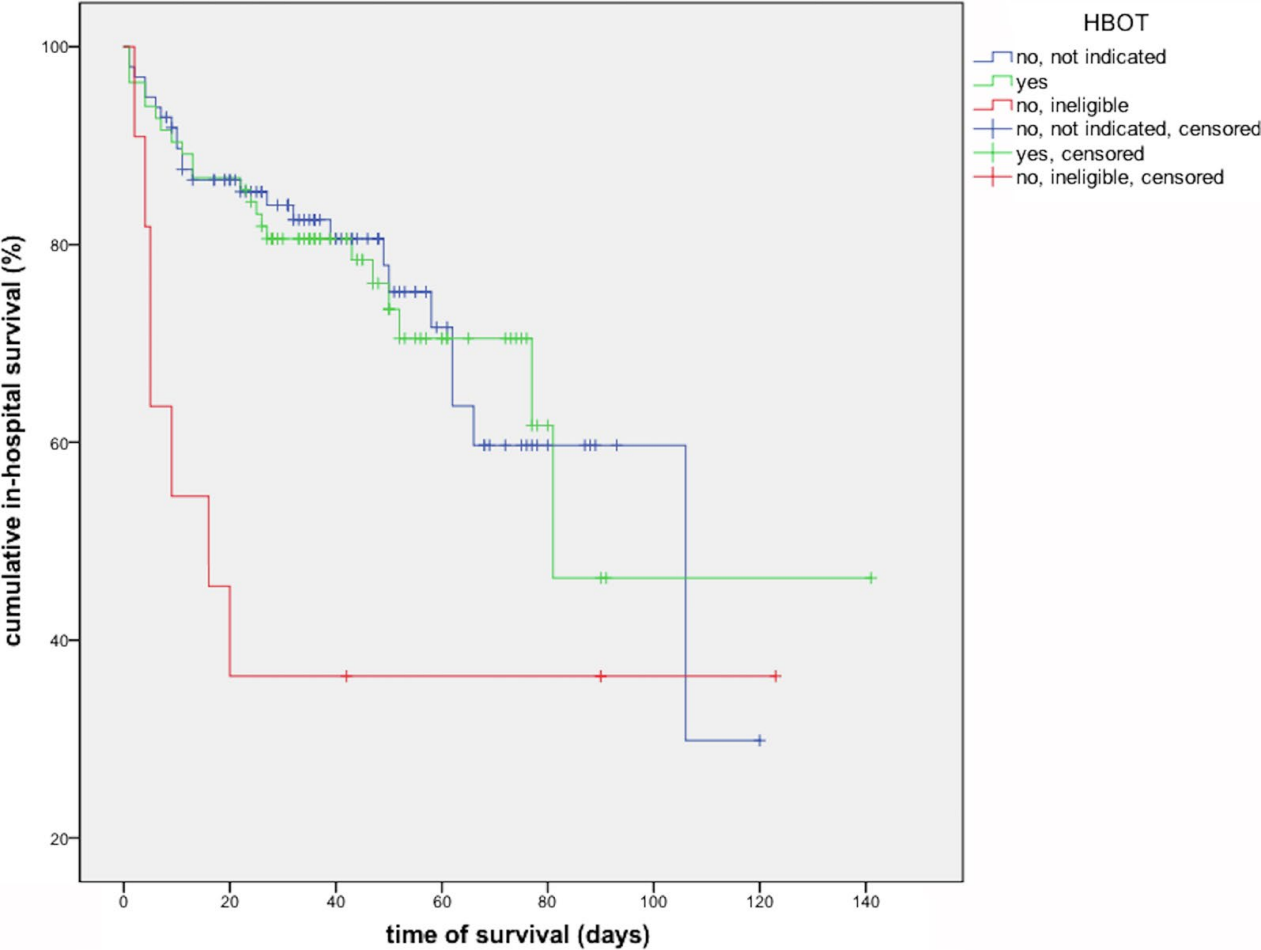
Kaplan–Meier curve showing cumulative in-hospital survival. Compared to patients without requirement of hyperbaric oxygen therapy (HBOT) and patients treated with HBOT, those ineligible for HBOT had significantly lower estimated survival times (*P* = 0.045 in log rank tests). Discharged patients were censored

In-hospital survival times were similar in patients without requirement of HBOT compared to HBOT patients, but shorter in the subgroup ineligible for HBOT (mean 82.5 vs. 91.8 vs. 50.3 days, median 106.3 vs. 81.7 vs. 16.5 days; Table 5, Fig. 4, *P* = 0.045 in log rank test).

**Table 5:** In-hospital survival times of patients with and without hyperbaric oxygen therapy (HBOT)

HBOT	Mean			Median		
	days	SD	95% CI	Days	SD	95% CI
Total cohort	90.5	6.6	77.5–103.5	106.0	23.6	59.8–152.2
HBOT subgroups						
No, not indicated	82.5	6.2	70.4–94.6	106.0	28.8	49.5–162.5
Yes^a^	91.8	10.5	71.2–112.4	81.0	n.d	n.d
No, ineligible	50.3	16.6	17.6–82.9	16.0	8.3	0.0–32.2

Mean and median in-hospital survival times were defined as days from admission to our or another hospital until death or discharge from our hospital

*CI* confidence interval, *n.d.* not determined, *SD* standard deviation

^a^In the subgroup with HBOT, the SD and 95% CI of the median in-hospital survival time could not be calculated due to lack of variability after the median

### Determinants on mortality according to multivariate regression analysis

The baseline multivariate logistic regression model adjusted for sex, age, localization of the NSTI, number of comorbidities and LRINEC showed an increased risk of mortality with rising age (odds ratio (OR) = 1.03, *P* = 0.017) and infection in a problem localization (OR = 2.88, *P* = 0.003; Table 6). When additional parameters were included into this model, ineligibility for HBOT (OR = 8.59, *P* = 0.005), pathogen detection in blood cultures (OR = 3.36, *P* = 0.002), complications (OR = 10.35, *P* < 0.001) and sepsis/organ dysfunction (OR = 19.58, *P* < 0.001) were associated with increased mortality, whereas a larger number of debridements (OR = 0.83, *P* < 0.001), VAC therapy (OR = 0.17, *P* < 0.001), defect closure with mesh graft (OR = 0.06, *P* < 0.001) and with flaps (OR = 0.09, *P* = 0.024) and a longer duration of the hospital stay (OR = 0.94, *P* < 0.001) were associated with a lower risk of mortality.

**Table 6:** Multivariate logistic regression models including potential determinants associated with mortality

	Characteristic	OR	95% CI	p
Baseline model^a^	Female	1.02	0.50—2.06	0.958
	Age, y	**1.03**	**1.01—1.06**	**0.017**
	Problem localization^b^	**2.88**	**1.44—5.77**	**0.003**
	Comorbidities, n	0.99	0.79—1.25	0.958
	LRINEC	1.00	0.89—1.13	0.992
Model 1	Debridements, n	**0.83**	**0.76—0.91**	** < 0.001**
Model 2	VAC therapy	**0.17**	**0.08—0.36**	** < 0.001**
Model 3	Mesh graft	**0.06**	**0.03—0.16**	** < 0.001**
Model 4	Flap	**0.09**	**0.01—0.73**	**0.024**
Model 5	Amputation	2.09	0.98—4.46	0.058
Model 6	HBOT, yes	1.06	0.49—2.30	0.884
	HBOT no, ineligible	**8.59**	**1.90—38.86**	**0.005**
Model 7	Positive blood culture	**3.36**	**1.57—7.20**	**0.002**
Model 8	Antibiotic groups, n	0.97	0.74—1.26	0.800
Model 9	ICU treatment	7.26	0.88—59.91	0.065
Model 10	Days in hospital	**0.94**	**0.93—0.96**	** < 0.001**
Model 11	Complications	**10.35**	**3.35—32.07**	** < 0.001**
Model 12	Infections	0.72	0.36—1.41	0.337
Model 13	Sepsis/organ dysfunction	**19.58**	**8.06—48.20**	** < 0.001**

*OR* Odds ratio, *CI* confidence interval, *FG* Fournier`s gangrene, *HBOT* hyperbaric oxygen therapy, *ICU* intensive care unit, *LRINEC* Laboratory Risk Indicator for NECrotizing fasciitis, *n* number, *NF* necrotizing fasciitis, *VAC* vacuum-assisted closure, *y* years

^a^The baseline model contained mortality as dependent variable and sex, age, problem localization, LRINEC and number of comorbidities as independent variables. In models 1–13, one additional parameter per model was integrated as independent variable. Age, number of comorbidities, number of debridements, number of antibiotic groups and days in hospital were included as continuous variables. The reference group for female was male. The reference category for "HBOT, yes" and "HBOT no, ineligible" contained patients who did not obtain HBOT because it was not indicated. Reference groups for all other parameters consisted of patients without the respective characteristic

^b^The category "problem localizations" comprised patients with NF or FG in the retroperitoneal area, head/neck region and/or in multiple localizations. Significant findings are highlighted in bold

## Discussion

### Mortality rates in NSTI

Our retrospective single-center study includes a relatively large, well-characterized cohort of patients with NF and FG with an in-hospital mortality rate of 27.6%. This rate is rather high compared to the recent literature. The largest systemic review and meta-analysis on adjunctive HBOT in NSTI, which comprised 48,744 patients, showed an in-hospital mortality rate of 9.8% [11]. A recent large prospective, multicenter study reported a 30-day mortality of 19.4% and a septic shock rate of 30% [12]. In our cohort, sepsis at admission was more common (68.8%). Treatment of NSTI in centers with a high annual caseload of these entities generally enhances the chances of favorable outcome, but on the other hand, these centers are prone to receive patients with higher disease severity due to higher expertise and availability of adjunct treatment options [13, 14]. These factors may have contributed to worse outcome in our cohort.

A recent systematic review of 109 studies with 6,051 patients revealed a significant reduction in mortality after the year 2000 [15], while this tendency was not found in subsequent study periods [14]. Improvement in intensive care with decreasing sepsis-related mortality [16] and progress in antibiotic treatment [17] are regarded as main factors contributing to better survival.

### Impact of surgical treatment on mortality

Delayed surgery is known to be associated with increased mortality due to NSTI [18], and debridement within 12 h after admission is explicitly recommended [15, 19]. Our HBOT patients and the subgroup ineligible for HBOT had timely debridements significantly more often than the group without need of HBOT. Altogether, only 47.9% of our patients received their first debridement within 12 h after admission to our hospital. However, one third had already obtained a debridement prior to transfer to our center. Outcome of transferred and directly admitted patients was similar, supporting early transport of patients with NSTI to specialized centers as recommended in prior work [13, 20].

VAC therapy and defect reconstruction were associated with a higher chance of survival. These observations should rather be interpreted as correlation than as causality, as defect conditioning and reconstruction require vital wounds and hemodynamic stability. The benefit of defect coverage is nevertheless evident, as it reduces the risk of complications related to large wounds and promotes mobilization, which is essential for convalescence. A larger number of debridements, including minor interventions for wound conditioning, and a longer hospital stay were both significantly associated with survival, which should be interpreted within this line of argumentation.

### Impact of HBOT

To our knowledge, this study is the first to stratify patients with NSTI into three groups according to indication and practicability of HBOT. The subgroup treated with HBOT had significantly worse initial conditions and greater disease dissemination and required more arduous treatment compared to patients without indication for HBOT. Nevertheless, rates of survival, defect coverage and hospital discharge without severe disability were similar in both groups, which points to a benefit from HBOT.

Even if HBOT is routinely used for treatment of NSTI in specialized centers worldwide, unequivocal scientific evidence for its benefits has not been provided yet. Many studies investigating its impact on outcome of NSTI demonstrated positive effects [21–29], whereas others did not show significant benefit [30–34]. Past systematic reviews provided only limited evidence on the clinical advantages of HBOT and had difficulties performing reliable meta-analyses due to shortcomings in the design and limited cohort sizes of included studies, high risk of confounding, substantial study heterogeneity and poor concordance of inclusion criteria [4, 5, 35, 36]. According to the latest meta-analysis, HBOT improves the odds for survival of NSTI [11].

In our study, ineligibility for HBOT was associated with increased mortality and significantly worse outcome despite early and frequent surgical debridements, extensive antibiotic therapy and intense supportive care, corresponding to results from a large multicenter database study [31]. The most common reason for withholding HBOT despite available infrastructure and highly experienced staff even after introduction of an in-house hyperbaric chamber was severe hemodynamic instability, e.g., due to septic shock. By contrast morbid obesity prevented HBOT only before the in-house chamber was established. Morbidly obese patients were younger, had less comorbidities and also survived more frequently, albeit commonly with severe physical impairment. As HBOT contributes to better oxygenation of insufficiently perfused fatty tissue, it could be speculated that obese patients benefit particularly from HBOT, although respective clinical evidence is lacking so far.

Diabetes mellitus is known to be one of the main risk factors for NSTI. Concordant with the literature [37, 38], almost half of our participants suffered from this comorbidity. Compared to nondiabetics, patients with diabetes were reported to have a higher risk of a severe disease course [39] and of limp loss due to their NSTI [37, 38]. Therefore, and due to diabetes-associated comorbidities potentiating the risk of adverse outcome, patients with diabetes and NF or FG may be preferentially considered for HBOT. The same applies to immunocompromised patients and patients with concomitant cardiovascular and metabolic diseases that may contribute to particularly severe and complicated disease courses and unfavorable outcome.

HBOT was shown to significantly reduce the risk of amputation in patients with NSTI in earlier studies [11, 24, 40], which encourages its use in patients with limb localization. In our cohort a major amputation of the lower limb was performed in 44 patients. These patients did not differ significantly from others with regard to HBOT, but the percentage of patients with limb loss appeared particularly high in the subgroup ineligible for HBOT.

Another subgroup expected to benefit particularly from HBOT according to the literature are patients with FG. A recent systematic review demonstrated overall mortality rates of 16.6% and 25.9% in patients with FG with and without HBOT [29]. We did no detect accordant differences in our cohort, possibly because of the limited number of patients with FG.

During the whole study period, only one complication possibly related to HBOT was recorded, i.e., a ruptured pulmonal cavern after the first HBOT session. This low rate of complications indicates high safety and good tolerability of HBOT. Therefore, this adjunctive treatment should be offered to patients with severe NF or FG whenever possible, in particular if they suffer from comorbidities which increase the risk of adverse outcome or if they are threatened by limb loss.

### Limitations

Due to the single-center design and the limited cohort size, generalizability of our findings may be restricted. However, characteristics of our study population and prognostic factors identified are well compatible with the literature [14, 41]. Although the cohort size is relatively large compared to other single-center studies, the study may be underpowered for assessment of certain impact factors on mortality and outcome. Some subgroups including the one ineligible for HBOT were small. Therefore, relevant risk factors for mortality may have been missed.

Owing to the retrospective study design, some clinical and laboratory parameters were not consistently available and data quality might be inferior to that of prospective studies. Comorbidities were documented in categories without assessing their severity. Use of a classification system for comorbid conditions like the weighted Charlson Comorbidity Index [14, 42] should be considered in future studies. Smoking habits were not consistently documented in the patient records, because many patients were too critically ill to take a detailed smoking history at admission and required long-term ventilation in the further course.

The fact that a large proportion of patients were transferred to our center implicates the possibility of confounding factors originating from transferring hospitals and the risk of selection bias towards patients who were fit enough for transport.

The use of different hyperbaric chambers during the study period could be another source of selection bias. The old HBOT chamber was not suitable for morbidly obese and severely unstable patients. After implementation of the new chamber in October 2017, morbid obesity ceased to pose a restriction and hemodynamically unstable patients could be treated with higher safety. Indeed, the number of patients treated with HBOT increased noticeably after implementation of the new chamber, and the percentage of patients with NF or FG who received HBOT raised from 43% before 2018 to 58% between 2018 and 2020.

### Strengths

We present a relatively large retrospective study which provides insight into mortality and outcome of patients with NF and FG treated in a large HBOT center under routine clinical conditions. Our study was performed in a Department of Plastic and Reconstructive Surgery with a certified Center of Wound Medicine and a highly specialized national HBOT center with long-time focus on NSTI and long-standing experience in their treatment. Diagnostic criteria of NF and FG as well as inclusion and exclusion criteria of the study were precisely defined, which is not always the case in retrospective studies on treatment of NSTI. The indication of HBOT was made according to standardized criteria independent of individual preferences of the surgeon or other team members. Clinical and laboratory parameters as well as details on the HBOT sessions, surgical procedures, antibiotic and supportive treatment and complications were documented accurately and comprehensively. Information on defect reconstruction, which is lacking in many other studies, was captured in detail and integrated into the analysis. The impact of various patient-, disease- and treatment-related factors on morbidity and outcome of NF and FG was assessed in multivariate regression models controlling for the key confounding factors.

Patients were stratified according to the indication, but also according to practicability of HBOT, which has not been done in other studies so far but reflects real medical care situations well and allows for a comparison of mortality and outcome close to clinical reality.

## Conclusions

We present a relatively large single-center study on mortality and outcome of an accurately characterized cohort of patients with NF and FG treated in a hyperbaric referral center, in which we identified several factors associated with increased mortality: higher age, affection of multiple or problem localizations, ineligibility for HBOT, positive blood cultures, complications and sepsis/organ dysfunction. In patients eligible for HBOT, our data point to a beneficial effect of this procedure. Our study was the first to stratify patients into three groups according to indication and practicability of HBOT, a classification reflecting the clinical situation well. Clearly, further evidence is required to verify our findings as well as the utility of HBOT for NSTI.

## Supplementary Information

**Additional file 1. Supplementary Table S1**. Characteristics of patients who were ineligible for hyperbaric oxygen therapy (HBOT).

## Data Availability

The datasets used and/or analyzed during the current study are available from the corresponding author on reasonable request.

## Abbreviations

FG: Fournier’s gangrene
HBOT: Hyperbaric oxygen therapy
ICU: Intensive care unit
LRINEC: Laboratory Risk Indicator for Necrotizing Soft Tissue Infections
m: Meters
NF: Necrotizing fasciitis
NSTI: Necrotizing soft tissue infections
OR: Operating room
qSOFA: Quick Sequential Organ Failure Assessment
VAC: Vacuum-assisted closure
