# Supplementary material for: Outcome of necrotizing fasciitis and Fournier's gangrene with and without hyperbaric oxygen therapy: a retrospective analysis over 10 years
Source: World J Emerg Surg. 2022 Aug 5;17:43. doi: 10.1186/s13017-022-00448-6 (PMC9356491; doi:10.1186/s13017-022-00448-6)
Supplement: Supplementary file 1 — Additional file 1. Supplementary Table S1. Characteristics of patients who were ineligible for hyperbaric oxygen therapy (HBOT). [file 13017_2022_448_MOESM1_ESM.docx]

**Additional file 1**

**Supplementary Table S1.** Characteristics of patients who were ineligible for hyperbaric oxygen therapy (HBOT)

|  | Characteristic | | | | | | | | | | | | | |
| --- | --- | --- | --- | --- | --- | --- | --- | --- | --- | --- | --- | --- | --- | --- |
| Patient | Sex | Age (y) | NF/FG | Localiza-tion | Comor-bidities, n | Comorbidities, kind | Time of treatment^a^ | Reason for ineligibility^b^ | VAC | AB, n | Complications | Mortality | Impair-ment |  |
| 1 | f | 52 | NF | lower extremity | 1 | diabetes | 04/2011 (old chamber) | obesity  (BMI 69.2) | yes | 7 | sepsis/organ failure | yes | n.a. |  |
| 2 | m | 78 | NF | lower extremity | 3 | diabetes, peripheral arterial occlusive disease, cardiac diseases^c^ | 11/2014  (old chamber) | hemodynamic instability | no | 1 | sepsis/organ failure, stump complication | yes | n.a. |  |
| 3 | m | 48 | NF | lower extremity, perineal | 2 | diabetes, renal disease | 08/2015 (old chamber) | obesity  (BMI 43.2) | yes | 9 | sepsis/organ failure, impaired wound healing, decubitus, thrombosis | no | severe |  |
| 4 | m | 49 | NF | lower extremity, perineal, trunk | 3 | diabetes, renal disease, arterial hypertension | 11/2015 (old chamber) | obesity  (BMI 44.5) | yes | 8 | sepsis/organ failure, impaired wound healing, stump complication, thrombosis | no | severe |  |
| 5 | m | 39 | NF | trunk | 2 | diabetes, arterial hypertension | 11/2016 (old chamber) | obesity  (BMI 51.5) | yes | 8 | sepsis/organ failure, impaired wound healing, decubitus | no | severe |  |
| 6 | m | 46 | NF | lower extremity | 2 | alcoholism, hepatic disease | 04/2018 (new chamber) | lung cavern | yes | 4 | none | no | none |  |
| 7 | m | 63 | NF | lower extremity, perineal | 4 | diabetes, peripheral arterial occlusive disease, aortic valve insufficiency, hepatic disease | 08/2018 (new chamber) | hemodynamic instability | no | 2 | sepsis/organ failure | yes | n.a. |  |
| 8 | m | 84 | NF | perineal, trunk | 4 | diabetes, renal disease, atrial fibrillation, arterial hypertension | 01/2019 (new chamber) | hemodynamic instability | no | 9 | sepsis/organ failure | yes | n.a. |  |
| 9 | m | 30 | NF | lower extremity, perineal, trunk | 2 | i.v. substance abuse, hepatic disease | 04/2019 (new chamber) | lung cavern | no | 6 | sepsis/organ failure | yes | n.a. |  |
| 10 | m | 35 | NF | trunk | 2 | renal disease, arterial hypertension | 06/2019 (new chamber) | hemodynamic instability | no | 4 | none | yes | n.a. |  |
| 11 | m | 52 | NF | lower extremity | 1 | atrial fibrillation | 02/2020 (new chamber) | hemodynamic instability | no | 3 | sepsis/organ failure | yes | n.a. |  |

^a^ The time of treatment initiation in our center is indicated as month and year. Before 10/2017, the center was equipped with an HAUX Starmed 2200/5,5 Multiplace Chamber located in 200 m distance to the hospital ("old chamber"). Since 10/2017, HBOT is performed in an in-house HAUX Starmed Quadro300-2300/3,3/ICU Multiplace Chamber ("new chamber"). ^b^ Main reason for ineligibility for HBOT. ^c^ Cardiac diseases included coronary heart disease, chronic heart failure, atrial fibrillation and presence of a pacemaker due to cardiac arrhythmia. AB: antibiotics; BMI: Body Mass Index in kg/m²; f: female; FG: Fournier's gangrene; m: male; n: number; n.a.: not applicable; NF: necrotizing fasciitis; y: years.
